# Assessing Providers’ Approach to Hypertension Management at a Large, Private Hospital in Kampala, Uganda

**DOI:** 10.5334/aogh.2513

**Published:** 2020-01-14

**Authors:** Aliza S. Green, Hayley M. Lynch, Rose Clarke Nanyonga, Allison P. Squires, Darinka D. Gadikota-Klumpers, Jeremy I. Schwartz, David J. Heller

**Affiliations:** 1 Department of Health System Design and Global Health, Icahn School of Medicine at Mount Sinai, New York, US; 2 Clarke International University, Kampala, US; 3 NYU Rory Meyers College of Nursing, New York, US; 4 Section of General Internal Medicine, Yale School of Medicine, New Haven, CT, US

## Abstract

**Background:**

Hypertension is increasingly prevalent in Uganda and its clinical management remains suboptimal across the country. Prior research has elucidated some of the factors contributing to poor control, but little is known about providers’ approaches to hypertension management and perceptions of barriers to care. This is particularly true in private health care settings – despite the fact that the private sector provides a substantial and growing portion of health care in Uganda.

**Objective:**

Our exploratory, pragmatic qualitative study aimed to examine the factors affecting the quality of hypertension care from the perspective of providers working in an urban, private hospital in Uganda. We focused on the organizational and system-level factors influencing providers’ approaches to management in the outpatient setting.

**Methods:**

We conducted interviews with 19 health care providers working in the outpatient setting of a 110-bed, private urban hospital in Kampala, Uganda. We then coded the interviews for thematic analysis, using an inductive approach to generate the study’s findings.

**Findings:**

Several themes emerged around perceived barriers and facilitators to care. Providers cited patient beliefs and behaviors, driven in part by cultural norms, as a key challenge to hypertension control; however, most felt their own approach to hypertension treatment aligned with international guidelines. Providers struggled to collaborate with colleagues in coordinating the joint management of patients. Furthermore, they cited the high cost and limited availability of medication as barriers.

**Conclusions:**

These findings offer important strategic direction for intervention development specific to this Ugandan context: for example, regarding culturally-adapted patient education initiatives, or programs to improve access to essential medications. Other settings facing similar challenges scaling up management of hypertension may find the results useful for informing intervention development as well.

## Introduction

Hypertension and other non-communicable diseases are growing public health concerns across Sub-Saharan Africa [1,2]. Deaths due to cardiovascular disease (CVD) rose by 81% between 1990-2013 in Sub-Saharan Africa, and the Pan-African Society of Cardiology (PASCAR) has identified control of hypertension as the highest priority for reducing CVD across Africa [3,4]. Uganda is no exception: the prevalence of hypertension among adults is approximately 20–25%, and control rates are consistently poor [5,6,7,8]. Limited resources, ill-equipped health facilities, and patients’ lack of awareness and adherence are key barriers to hypertension control in Uganda [9,10].

Prior research on health facilities’ capacity for hypertension control in Uganda has identified barriers including scarce medications and diagnostic equipment, inadequate training of personnel, insufficient knowledge and experience of clinicians in the management of CVD and hypertension, and a lack of guidelines or resources for hypertension management [11,12]. The knowledge, attitudes, and practices of physicians and nurses regarding hypertension in Uganda remains largely unstudied, especially in private care settings – despite the fact that the private sector provides a substantial portion of health care in Uganda. According to a household survey conducted in 2006, approximately 46% of Ugandans who sought health care did so from a private clinic, compared to 22% who sought from a government health unit [13]. A similar survey conducted in 2010 yielded comparable results, with private health care dominating the ambulatory care landscape [14].

In this study, we explore factors affecting the quality of hypertension care from the perspective of providers working in a private hospital. We were specifically interested in capturing their insights into the organizational and system-level factors that influence their approaches to hypertension management in the outpatient setting.

## Materials and Methods

### Study design

A pragmatic qualitative study design underpinned our approach to the study. Pragmatic studies, a category of generic qualitative descriptive research designs, seek to generate findings which are immediately translatable and applicable to the context where the study occurred [15]. The hospital’s administration was a stakeholder in the study’s design, which aimed to generate results to inform improvements to care delivery in this setting.

### Theoretical framework

**Figure 1:**
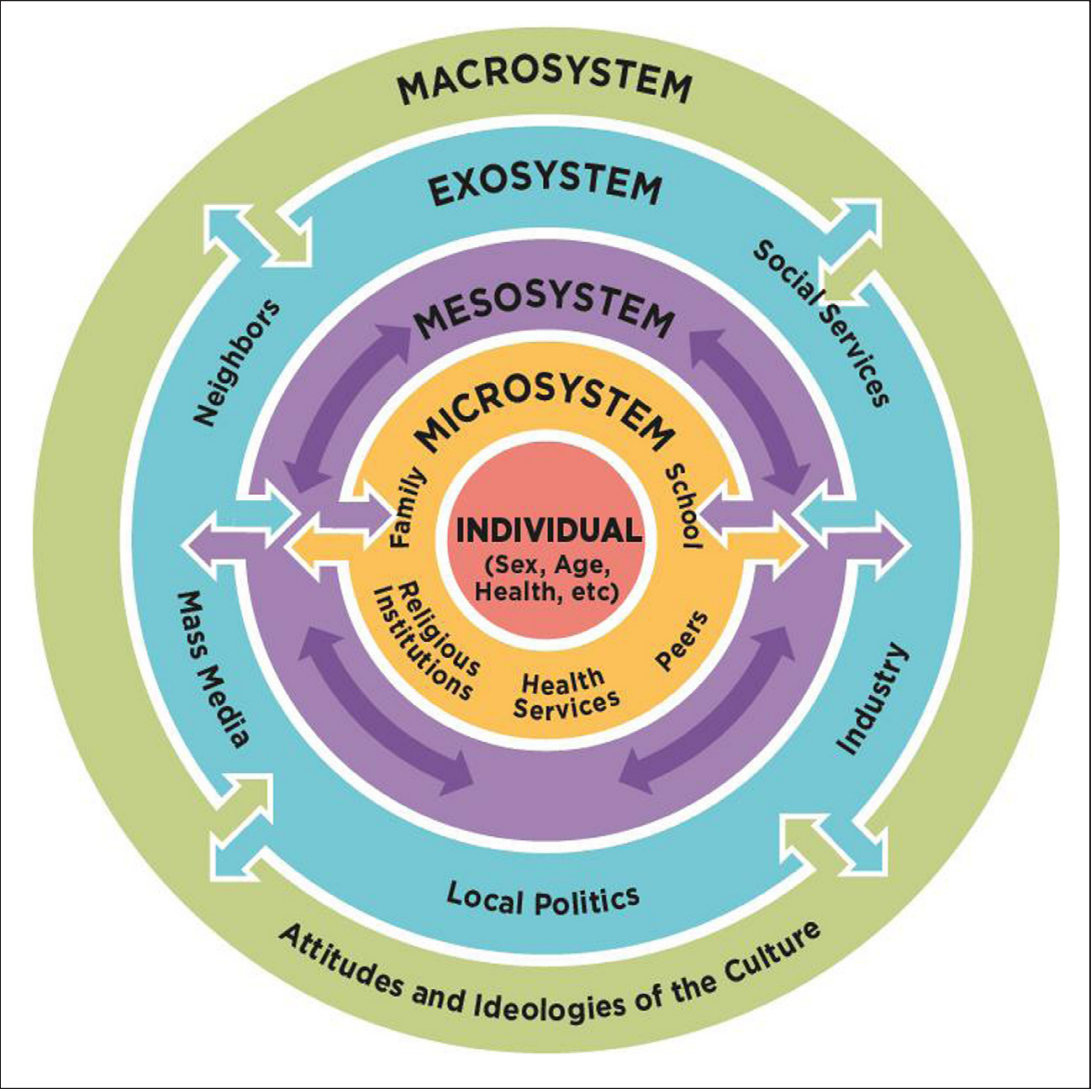
The Social-Ecological Model of Behavior Change [16,17]. Source: National Academies of Science, Engineering, and Medicine *Preventing Bullying Through Science, Policy, and Practice* (2016), as adapted from Bronfbrenner (1979).

We employed the social-ecological model, which provides a conceptual framework for understanding the complex and interrelated effects of an environment on individual behavior [16,17]. It comprises five nested levels – the individual, interpersonal, institutional, community and socio-cultural – corresponding to layered systems of influence (Figure 1). The framework provided a structure from which we could explore how providers’ hypertension practices are influenced by their context.

### Sample

We conducted our study at a large, 110-bed urban private hospital in Uganda. Study participants were recruited from the group of providers working in the outpatient setting between June and August 2017. Study locations included the outpatient department, specialist clinic, and cardiology clinic, and providers sampled included nurses, general practitioners (physicians with one year of post-graduate internship), general physicians (physicians with post-graduate specialist training in internal medicine), and cardiologists.

### Recruitment and data collection

We selected participants based on their degree of contact with hypertensive patients in the outpatient setting. Inclusion criteria designated English proficiency as a requirement for practical reasons (neither interviewer spoke other languages fluently), but this did not limit participation because all providers approached were fluent English speakers. We purposively sampled to ensure that participants had a diversity of work experience, gender, and training level. We recruited participants both by public announcement at a continuing medical education meeting and then individually based on staff lists or word of mouth. We sought to recruit between 15 to 20 providers, as that sample size would allow us to achieve data saturation [18].

The semi-structured interview guides were designed to assess providers’ approach to hypertension management: specifically, screening, diagnosis, treatment, follow-up, and use of the electronic medical record (EMR). We also asked about perceived barriers to hypertension care and hospital-specific challenges. The content of our qualitative guides was informed by initial findings from concurrent quantitative research, which investigated rates of hypertension prevalence, diagnosis, and control in the same setting. For example, observed inconsistencies in electronic recordings of blood pressures guided our questions about screening protocols and utilization of the EMR. We pilot-tested the interview guide with a Ugandan physician employed by the hospital to ensure that questions were clear, consistent with cultural norms, and appropriate for the clinical setting. We designed four research guides tailored to each provider type (e.g. general practitioner versus cardiologist); each was specific to the providers’ responsibility in managing hypertensive patients and their role in the context of an interdisciplinary hospital system. Following our first interview with a specific clinician type, we updated the guide informed by their responses and suggestions. We continued to iteratively update the guides’ content throughout the interview period based on emerging trends and provider feedback. The final interview guides are included as appendices.

Two researchers [ASG and HML] conducted participant interviews in English after obtaining written consent. Interviews were 30–50 minutes in length and were digitally recorded. Research team members transcribed the interviews, with transcription quality checked by a separate team member.

### Analysis

We developed themes and codes for the study by directed content analysis, a method that uses predefined categories and concepts to structure the analytic process [19]. We based our codes and themes on the social-ecological model as described above, which allowed us to explore the relationships between individual providers and their broader interpersonal, cultural, and societal context. The iterative process facilitated a holistic analysis of hypertension management from the patient through the system level.

To conduct the coding process, three researchers [ASG, HML, DGK] used a sample of selected transcripts to independently generate codes, and then shared these codes with colleagues [DJH, APS, RNC] to iteratively generate a standardized codebook. Using this refined codebook, one researcher [ASG] utilized NVIVO software (version 11) to re-code all interviews and generate reports. We then analyzed these reports for patterns and emerging themes, with final themes and categories generated by team consensus.

### Ethical Review

The study design, data collection tools, and consent forms were approved by the Mount Sinai Institutional Review Board, Clarke International University Research Ethics Committee, and the Uganda National Council of Science and Technology.

## Results

Our final sample included 4 nurses (N), 8 general practitioners (GP), 4 general physicians (PH), and 3 cardiologists (C) for a total of 19 participants. Demographic data on participants is displayed in Table 1. Five themes emerged from our interviews as discussed below. They reflect a complex practice environment shaped by interprofessional dynamics, patients’ cultural beliefs and level of health literacy, and governmental prioritization of health care resources.

**Table 1:** Interview Participant Demographics.

Gender	n (%)

Male	8 (42%)
Female	11 (58%)
**Provider Type**	

General Practitioner	8 (42%)
General Physician	4 (21%)
Cardiologist	3 (15%)
Charge Nurse	2 (11%)
Triage Nurse	2 (11%)

### Patient self-management

All providers cited patient-centered factors, particularly medication and lifestyle non-adherence, as central challenges to hypertension management. Many physicians explained that patients do not feel sick, and thus see no reason to take medications: ‘[It] is within their means to adhere. But for something that is not causing any symptoms, becomes very tricky to enforce’ (C2). Relatedly, patients struggled to accept that chronic conditions require long-term medications. One provider explained, ‘So, that’s a problem we always get. Them to accept that they have a problem that is going to need life-long treatment. And they are very resentful to that’ (GP4).

Providers also described cultural expectations about weight and dieting as challenges to management. As one provider described:

Some people, for example, believe that when you’re obese it’s a sign of money. So they can’t adjust despite you advising them that we can’t manage your pressure as much as we give you drugs when you’re still overweight…and so they will think, when I lose weight, people know me by this size, when I lose weight people will think I’m, either HIV positive or bankrupt (GP3).

Providers also felt that patients were skeptical about “Western” medicine, and preferred alternative, herbal-based treatments found in the community. Other factors included lack of understanding about hypertension and its consequences, denial about their condition, and the inability to afford medications and other treatments.

### Providers’ practices were consistent internally and with international guidelines

Participants were asked about their approach to screening, diagnosis, patient education, follow up, monitoring for complications, and treatment of hypertension. All interviewed nurses, who were responsible for taking and recording pressures, reported that every patient regardless of presenting complaint had their blood pressure taken at triage before visits. This was confirmed by general practitioners and physicians, who would not see patients until vitals had been obtained and logged. The one exception was the cardiology clinic, in which physicians took blood pressure measurements themselves. Providers across specialties reported similar thresholds and criteria for a hypertension diagnosis, and generally agreed that a diagnosis of hypertension required an elevated blood pressure reading on more than one occasion. Most considered assessment for secondary causes of hypertension (e.g., renal disease) a routine part of diagnosis:

Our next step [after diagnosis] is to evaluate the cause. Much as we know that 95% the cause is not identified, but we will evaluate for the cause… by doing some laboratory investigations, further physical examinations, and then appropriate radiological investigations (GP3).

Providers similarly described evaluations for complications of hypertension as standard-of-care. Serum lipids, glucose, renal and liver function tests, complete blood count, echocardiograms, and electrocardiography were cited as standard follow-up tests for new diagnoses.

Nurses and physicians perceived patient counseling as crucial to effective hypertension management and made patient education a priority of care. All clinicians mentioned providing diet and exercise counseling, and several regularly educated their patients about weight reduction, smoking cessation, reduction of alcohol intake, medication adherence and the long-term risks of hypertension. Most providers recommended lifestyle modification as initial hypertension therapy before starting medications, except in severe cases. Physicians prescribing practices were highly consistent: they chose medications based on patients’ comorbidities and end-organ damage, as well as drug cost and medication availability. Several practitioners and specialists additionally identified patient ethnicity as key factor in decision-making. When prompted about utilization of hypertension medications in different patient populations, one provider noted, ‘The only difference I find is that beta-blockers are more effective in Caucasians. than in our natives, our Africans… the beta-blocker alone will hardly make an impact on Africans with hypertension’ (GP4).

Practitioners and specialists generally believed hypertension control rates in their patient population were high but differed in their perception of what constituted a “controlled” patient. Most providers estimated control rates of 65–95% among patients in their care. Only two providers estimated lower control rates: 40% and 50% respectively. The participants who estimated high control rates more often cited patient adherence as a major limitation to blood pressure control.

Numerous providers, particularly specialists, relied on international guidelines to inform their diagnostic and treatment practices. Five participants reported utilizing the *Joint National Committee on Prevention, Detection, Evaluation, and Treatment of High Blood Pressur*e (JNC) guidelines, and other mentioned guidelines included those created by the United Kingdom’s *National Institute for Health and Care Excellence* (NICE), the *American Association for Family Physicians* (AAFP), the *American Heart Association*, the *Uganda Ministry of Health*, and *Uganda Clinical Guidelines*.

### Challenges with care coordination, interprofessional collaboration and communication

Providers were confident and competent at individual-level hypertension management but lacked clarity on how to operate efficiently within the hospital system. For example, providers disagreed on which personnel and departments should manage hypertensive patients:

GP3: ‘Though the protocol is every patient with hypertension should be transferred to a physician.’

PH2: ‘I personally do not like chronic patients to be followed up in the OPD [outpatient department]. A lot of patients still come to the OPD with hypertension, diabetes, and so on because of convenience, but then what tends to happen is that they end up seeing different doctors, which to me is not an ideal situation. So I tend to channel them to the specialist center.’

GP10: ‘When controlled, there’s no complications… we see them, then we will ask them to come back after one month. So in case there are complications that we are not able to handle, we refer them to specialist.’

PH3: ‘So, if the patient’s blood pressure is okay and they [General Practitioners] don’t see any big issue going on, they don’t have to call me.’

There was also no uniform system for transferring referred patients’ information between departments. General practitioners in the outpatient department relied on the hospital’s electronic medical record in conjunction with a paper-based system; other physicians and specialists used a different paper chart in clinic. As such, providers lacked a single platform to track each patient’s progress, as articulated by a physician member of the hospital administration:

Sometimes we get patients who come in and they say they want a drug refill. So, somebody has not taken care to see, when was their last renal function, what’s happening with all that, what’s happening with eGFR and then before you know it in 2 years’ time, you look and you see somebody’s renal function actually deteriorated. So, that’s my main concern. Just making sure that we are not having that end organ damage as a result of no proper follow up. Because when they come to the [specialist] clinics, they have the book…yeah they have the follow up book so you can easily look back and see, when did this person have their lipid profile? When did they have their renal function? While when they come to the [outpatient department], they have some pieces of paper which are then taken away and stored, and the patient may not come back with their copy from the last time. So, you ask them when was the last test done. And then you have to go and check, it takes time. So people will omit some of those things because of the time (PH2).

Awareness and utilization of hospital guidelines was also highly variable. Often, one provider would cite a specific requirement or protocol – around diagnosis, treatment, referral or follow up practices – that other providers would contradict or not mention. Multiple providers, when prompted, denied any knowledge or use of guidelines specific to their hospital setting. However, most participants demonstrated enthusiasm for the development and implementation of hospital-wide protocols: ‘maybe if we could have protocols, SOPs [standard operating procedures], that would help…So that if I saw a patient or any other person saw a patient, we should come to the same conclusion about how to go about it’ (C2).

Generally, these observed inconsistencies in care coordination were not perceived as major impediments to effective care delivery. For example, when providers were probed specifically about challenges related to medical record-keeping, they reported frustrations with the EMR and acknowledged its limited potential for exchanging patient information. However, when asked more generally about barriers to hypertension care, no providers volunteered the EMR system as a barrier.

### Existing systems challenge efficient hypertension management

Providers differed regarding which factors within the hospital system hindered hypertension care. Some providers believed that treatment cost rarely compromised care, but others considered the price of care (particularly medications and lab testing) to be a central barrier to effective treatment of hypertensive patients. One provider reported that his patients could often afford a visit but not the follow-up labs and tests, limiting his ability to optimize their management.

Similarly, some providers believed that the pharmacy stocked the most important hypertension medications adequately but lacked fixed-dose combination or specialty medications. Some complained that even basic medications were not available while others felt the pharmacy supply was entirely satisfactory. Several providers stated that generic, imported drugs stocked by the pharmacy were not effective:

But the challenge is the majority of the drugs, Indian drugs, Chinese drugs, the majority of the drugs stop. Sometimes you give, [and] you think reading of the blood pressure would be brought down by nifedipine alone… and the [blood pressures] are still elevated. You are forced to introduce a second drug. Yet, one drug would be enough. When they start to swallow the drug from Europe, they end up taking just half a dose and their pressures are no longer elevated. So one of the challenges is fake drugs. And we’ve been complaining [about that] (GP3).

Providers also noted that their patients shared the same concern: some patients even refused treatment with non-European medications.

### The intersection of cultural norms, care seeking behaviors, and NCDs

Providers also cited socio-cultural factors as barriers to hypertension care delivery. Providers frequently complained about the absence of “wellness checks” in Uganda. Their patients were often only identified as hypertensive incidentally at unrelated visits, frequently late into their disease course and sometimes with end-organ complications. Many providers attributed this absence to a lack of effort from the media to educate the public about non-communicable diseases:

You have to be aware that you need the health checkup. Radio stations help in trying to advertise about health issues, sensitizing people. They are mostly, they are more like right now into cancers, which are prostate cancer, breast cancer, cervical cancer, is the campaign that they are really having now on health issues. But the issues about blood pressure and diabetes are not information tackled by the radio station. I think there’s a gap there, maybe you could try and make use of that (GP4).

Similarly, providers accused the government of failing to prioritize education and screening for chronic illness: ‘Our government has not taken up the initiative of educating people. It’s the NGOs… the churches, there are hospitals doing outreach… but the government does not. It’s not doing it the way it does it for HIV and other things’ (GP8). When prompted for suggestions to improve hypertension care, several providers mentioned societal solutions: increase public awareness, screening opportunities, and wellness checks and pressure the government to provide education and funding towards these ends.

## Discussion

To our knowledge, our study is the first to explore the approaches of urban private providers in Uganda to hypertension management, and the barriers they perceive as limiting hypertension control. The social-ecological model as a guiding framework helped us situate the provider responses in a layered context that reflected the “real world” challenges of implementing hypertension management programs in urban Uganda: their own behaviors; patients’ behaviors; hospital system challenges; and socio-cultural challenges.

Our study findings suggest that there are several barriers to effective hypertension care delivery: limited patient adherence, inconsistent referral systems, financial and logistical barriers, and lack of public awareness. They also indicate several facilitators of care, including provider knowledge of validated hypertension control strategies and a willingness to embrace new systems and protocols for care.

Although their views were often diverse, providers repeatedly cited extrinsic barriers (patient adherence, hospital systems, and socio-cultural factors) to hypertension care rather than barriers within their direct control (lack of skills or knowledge, inter-provider communication, or coordination). This finding is new in the Ugandan setting, but consistent with prior literature elsewhere. A systematic review of global quantitative and qualitative studies on barriers to hypertension control found that providers were more likely to cite health system barriers and social influence factors than individual capability barriers [20]. However, only 14 of the 69 included studies came from low- and middle-income countries. Our study suggests that this trend may be more common amongst providers in low-and-middle-income countries than was previously understood. Possible explanations for this result – and providers’ lack of self-criticism in general – include a lack of opportunities for providers to share and reflect on their practices; norms surrounding criticism of medical professionals; and reluctance to express doubt or disapproval of practices in the presence of external observers. Alternatively, providers may be unaware of their actual hypertension control rate, or of expectations for their clinical performance. Additional exploration, including quantification of blood pressure control rates, is warranted to corroborate these findings and understand whether they inform any clinician self-assessment bias.

Research to date suggests Ugandan patients’ beliefs and experiences regarding hypertension self-management – including incomplete disease knowledge, and skepticism regarding medication use – align with the impressions of providers we describe above [21,22]. Similarly, prior research demonstrates that providers’ concerns about adherence are well-founded given low adherence rates across Uganda [23]. Programs to inform and educate patients regarding the benefits of medication adherence and lifestyle change may be useful in the Ugandan private and public sector, as they have been in other settings [24,25,26,27,28]. Nurse-led self-management interventions are particularly promising, as nurses are the most numerous healthcare professionals in Uganda and research suggests that nurse-led interventions can improve hypertension control in outpatient settings in Uganda [29].

Such programs require reliable and consistent access to quality hypertension medications and services, factors which currently represent major barriers to care. Providers’ concerns regarding inconsistency in generic drug quality requires specific attention, especially as the World Heart Federation and PASCAR advocate for use of generic anti-hypertensives on their roadmaps to reduce hypertension and CVD deaths in Africa [3,30]. Future work in this realm might involve follow-up interviews with providers and patients, studies of pharmacy inventories, and supply-chain analysis to identify lapses in medication quality and accessibility. Broader strategies to increase public awareness and outreach, facilitate blood pressure screening, and encourage routine health check-ups similarly merit exploration.

A recent systematic review in low-and-middle-income countries found that poor quality (more than poor availability) of healthcare services contributed excessively to patient morbidity and mortality, particularly among cardiovascular-related deaths [31]. Care coordination challenges – such as inconsistent record-keeping, referral, and follow-up protocols – are part of those same quality issues cited in the review and will pose challenges to private facilities with diverse hypertension care pathways [32].

Targeted strategies to improve inter-provider communication, enhance record keeping, and standardize procedures might help ameliorate the challenges to care identified in this study. A clinical pathway for hypertension management, including indications for referrals, processes and mechanisms for information-sharing between providers, and guidelines around hypertension screening, diagnosis and treatment is one place to start. Providers’ stated enthusiasm for revised protocols – and the hospital’s current initiative to revise clinical protocols and update the EMR – suggest that this intervention would be both welcome and beneficial. This guideline would require adaptation to the Ugandan setting, as literature suggests that some aspects of U.S.-based CVD guidelines are unrealistic in low-and-middle-income populations and should be adapted accordingly [33]. Ideally, as processes are clarified and standardized for providers, the system will become more easily navigable by patients; in this way, addressing provider and hospital-level factors has the added effect of addressing the barrier that providers believe to be most relevant – patient behavior.

### Limitations

The generalizability of our findings is limited by the single site of the study: a large, private, urban hospital with a unique patient population (majority insured, middle upper class), a diversity of provider types (nurses, general practitioners, and specialists) and readily accessible services (in-hospital laboratory, full pharmacy). Our data collection and coding team comprised mostly non-Ugandan researchers, and all interviews were conducted in English. Thus, the research team’s prior beliefs regarding appropriate hypertension care – including the perceived efficacy and necessity of protocols and EMRs – may have biased our interpretation of the findings. As mentioned previously, interviews conducted by external observers may also have influenced providers’ responses, particularly those about their colleagues’ and their own clinical practices and decision-making. Nevertheless, our findings are consistent with prior data from high-income and limited low-and-middle-income settings as detailed above.

## Conclusions

This study, among the first in Uganda to evaluate private-sector hypertension care in a resource-limited setting dominated by private health providers, offers strategic directions to develop interventions that can improve the quality of hypertension management by providers and address patient adherence issues. Our results suggest that future interventions designed to account for patient, provider, organizational and systemic factors may have the best chance to succeed.

## Additional Files

The additional files for this article can be found as follows:

10.5334/aogh.2513.s1 Appendix A. Qualitative Interview Guide for Nurses.

10.5334/aogh.2513.s2 Appendix B. Qualitative Interview Guide for General Practitioners.

10.5334/aogh.2513.s3 Appendix C. Qualitative Interview Guide for General Physicians.

10.5334/aogh.2513.s4 Appendix D. Qualitative Interview Guide for Cardiologists.
