# Supplementary material for: Assessing Providers’ Approach to Hypertension Management at a Large, Private Hospital in Kampala, Uganda
Source: Ann Glob Health. 2020 Jan 14;86(1):5. doi: 10.5334/aogh.2513 (PMC6966335; doi:10.5334/aogh.2513)
Supplement: Appendix A. — Qualitative Interview Guide for Nurses. [file agh-86-1-2513-s1.pdf]

Appendix A: Qualitative Interview Guide for Nurses

1. What clinic do you work in? Can you define your role?
2. Are there ever times when you cannot check a patient's blood pressure in triage?
3. Which arm is pressure taken from?
4. What position is the arm in?
5. Are patients always sitting when you take blood pressure?
6. How many blood pressure readings are done for each patient? (probe: if its high on first read, what next)

For nurses working in the outpatient department:

7. Where do you record the blood pressure?
8. Are you always able to record in all of those places?
9. With the old EMR, were there any challenges recording blood pressure?
    a) If the EMR was down, what would you do?
    b) If you had to record it in the log book, was information transferred later into EMR? How often?
10. With the new EMR, are there any challenges recording blood pressure?
    a) If it doesn't work, what do you do? Will information be transferred later?
11. If you take more than one blood pressure of a patient in a visit, how many of those readings are recorded? Where?
12. If a patient's pressure is high, what do you say to them? How do you explain high pressure? Do you discuss approaches to management/control with patients?
13. If a patient's pressure is very high, do you ever send them to a doctor immediately? Do you ever send them to the emergency department?
14. Do you have any suggestions/feedback for the study?

For nurses working in the specialist center:

7. Where do you record the blood pressure   Are you always able to record in all of those places?
8. Are you currently putting patients' blood pressure's into the EMR?
    a) What other information is going into EMR?
    b) Are there any challenges using EMR?
    c) If it doesn't work, what do you do? Will information be transferred later?
9. In the past, were you putting patients' blood pressure's into the old EMR?
    a) If the EMR was down, what would you do?
    b) If you had to record it in the log book, was information transferred later into the EMR? How often?
10. Do doctors ever ask you to access information in the EMR for them?
11. If a patient's pressure is high, what do you say to them? How do you explain high pressure? Do you discuss approaches to management/control with patients?

12. If a patient’s pressure is very high, do you ever send them to a doctor immediately? Do you ever send them to the emergency department?
13. Do you have any suggestions/feedback for the study?
