# Supplementary material for: Assessing Providers’ Approach to Hypertension Management at a Large, Private Hospital in Kampala, Uganda
Source: Ann Glob Health. 2020 Jan 14;86(1):5. doi: 10.5334/aogh.2513 (PMC6966335; doi:10.5334/aogh.2513)
Supplement: Appendix C. — Qualitative Interview Guide for General Physicians. [file agh-86-1-2513-s3.pdf]

Appendix C: Qualitative Interview Guide for General Physicians

1. Which clinics do you work in when you're at this hospital?
2. How do you know what a patient's blood pressure is when they come into clinic? (probe: who takes it, where is it recorded, is it always possible to record)
3. How often are your patients' blood pressures checked? (probe: if infrequent, will you take the blood pressure?)
4. How do you make a hypertension diagnosis?
5. How does the diagnosis of hypertension changed based on different factors (probe: age, sex, comorbidities)?
6. Where do you record a diagnosis of hypertension? How is it coded?
7. What are the guidelines around hypertension at this hospital? (probe: how are you trained in them, would you prefer more/less guidelines)
8. If you diagnose a patient with hypertension, what are your next steps? (probe: assessment of secondary causes)
9. What is your treatment plan for a patient with hypertension?
    a) How do you decide whether to prescribe medication for a patient with hypertension?
    b) When you prescribe medication, how do you decide which medication to give?
    c) Is diet/exercise counseling part of your treatment plan?
10. How do you explain a diagnosis of hypertension to a patient?
11. How well do you think patients understand hypertension?
12. How do you record and code prescriptions?
13. How frequently do you schedule follow-up appointments for patients with hypertension?
14. How often are your hypertensive patients lost to follow-up?
15. How are you monitoring changes in blood pressure for patients? Where are these changes being tracked?
16. What is your relationship with General Practitioners, relating to the division of hypertension management at this hospital? (probe: referral processes)
17. Do you believe hypertension is a large problem in your patient population?
18. How many of the hypertensive patients have achieve control? How do you define control?
19. Have you faced any challenges in providing hypertension care to your patients?
    a) Do you have sufficient time with patients to provide hypertension care?
    b) Do you have adequate resources to provide hypertension care (probe: nurse support, reliable medication access, necessary tools/equipment)
    c) Is patient non-compliance an issue? If so, what factors are making patients not comply?
    d) Does the EMR/record keeping system effect hypertension management here?
20. Is there anything else you would like to share or discuss with respect to hypertension care? Do you have any suggestions/feedback on this study? (probe: questions we should be asking).
