# Supplementary material for: Assessing Providers’ Approach to Hypertension Management at a Large, Private Hospital in Kampala, Uganda
Source: Ann Glob Health. 2020 Jan 14;86(1):5. doi: 10.5334/aogh.2513 (PMC6966335; doi:10.5334/aogh.2513)
Supplement: Appendix D. — Qualitative Interview Guide for Cardiologists. [file agh-86-1-2513-s4.pdf]

Appendix D: Qualitative Interview Guide for Cardiologists

1. How do you know what a patient's blood pressure is when they come into clinic? Where is it recorded?
2. If patients are referred to you, how do you access their past medical history? (probe: use of EMR, use of physical records)
3. Are you ever the one to make a hypertension diagnosis?
    a) If yes, how do you do it?
4. Under what circumstances are patients with hypertension referred to you? By whom?
5. What is your treatment plan for a patient with hypertension?
    a) When you prescribe medication, how do you decide which medication to give?
    b) Where do you record the prescriptions?
6. How frequently are you scheduling follow up appointments for patients with hypertension?
    a) At those follow up appointments, which follow up tests are you doing?
7. How frequently are your hypertensive patients lost to follow up?
8. Approximately how many of the hypertensive patients you have achieve control?
9. How do you define control?
10. What is your relationship with the other providers at this hospital as far as managing hypertensive patients? How do you share information with them about patients?
11. What are the most common hypertension-related complications you're seeing?
12. Do you work anywhere other than this hospital?
13. If yes, have you noticed any differences between this hospital and other hospitals in terms of hypertension rates? In terms of hypertension-related complications? In terms of hypertension control?
14. Are there any protocols specific to this hospital that you utilize while practicing here?
15. Have you faced any challenges at this hospital in providing hypertension care to your patients?
    a) Is patient non-compliance an issue?
        i. How do you assess compliance?
        ii. What reasons, do you think, make patients not comply?
    b) Do you feel you have adequate time/resources to provide hypertension care?
    c) Do you encounter issues with patients' ability to afford diagnostic tests?
16. Do you have any suggestions/feedback for this study? (probe: questions to be asking, information to be gained, etc).
